# Correction: Kernel Density Surface Modelling as a Means to Identify Significant Concentrations of Vulnerable Marine Ecosystem Indicators

**DOI:** 10.1371/journal.pone.0117752

**Published:** 2015-01-21

**Authors:** The PLOS ONE Staff

## Notice of Republication

This article was republished on December 12, 2014 to correct errors in the figure captions that were introduced during the typesetting process. Please download this article again to view the correct version. The originally published, uncorrected article and the republished, corrected article are provided here for reference.

## Supporting Information

S1 File Originally published, uncorrected article. (PDF)

S2 File Republished corrected article. (PDF)
