# Supplementary material for: Correction: Kernel Density Surface Modelling as a Means to Identify Significant Concentrations of Vulnerable Marine Ecosystem Indicators
Source: PLoS One. 2015 Jan 21;10(1):e0117752. doi: 10.1371/journal.pone.0117752 (PMC4301816; doi:10.1371/journal.pone.0117752)
Supplement: S2 File — (PDF) [file pone.0117752.s002.pdf]

OPEN ACCESS Freely available online

PLOS | ONE

Kernel Density Surface Modelling as a Means to Identify Significant Concentrations of Vulnerable Marine Ecosystem Indicators

Ellen Kenchington[1]*, Francisco Javier Murillo[1,2], Camille Lirette[1], Mar Sacau[3], Mariano Koen-Alonso[4], Andrew Kenny[5], Neil Ollerhead[4], Vonda Wareham[4], Lindsay Beazley[1]

1 Bedford Institute of Oceanography, Department of Fisheries and Oceans, Dartmouth, Nova Scotia, Canada, **2** Departamento de Zooloxía e Antropoloxía Física, Facultade de Bioloxía, Universidade de Santiago de Compostela, Santiago de Compostela, Spain, **3** Instituto Español de Oceanografía, Centro Oceanográfico de Vigo, Programa de Pesquerías Lejanas, Vigo, Spain, **4** Northwest Atlantic Fisheries Centre, Department of Fisheries and Oceans, St. John's, Newfoundland and Labrador, Canada, **5** Centre for Environment, Fisheries and Aquaculture Science, Lowestoft, Suffolk, United Kingdom

Abstract

The United Nations General Assembly Resolution 61/105, concerning sustainable fisheries in the marine ecosystem, calls for the protection of vulnerable marine ecosystems (VME) from destructive fishing practices. Subsequently, the Food and Agriculture Organization (FAO) produced guidelines for identification of VME indicator species/taxa to assist in the implementation of the resolution, but recommended the development of case-specific operational definitions for their application. We applied kernel density estimation (KDE) to research vessel trawl survey data from inside the fishing footprint of the Northwest Atlantic Fisheries Organization (NAFO) Regulatory Area in the high seas of the northwest Atlantic to create biomass density surfaces for four VME indicator taxa: large-sized sponges, sea pens, small and large gorgonian corals. These VME indicator taxa were identified previously by NAFO using the fragility, life history characteristics and structural complexity criteria presented by FAO, along with an evaluation of their recovery trajectories. KDE, a non-parametric neighbour-based smoothing function, has been used previously in ecology to identify hotspots, that is, areas of relatively high biomass/abundance. We present a novel approach of examining relative changes in area under polygons created from encircling successive biomass categories on the KDE surface to identify "significant concentrations" of biomass, which we equate to VMEs. This allows identification of the VMEs from the broader distribution of the species in the study area. We provide independent assessments of the VMEs so identified using underwater images, benthic sampling with other gear types (dredges, cores), and/or published species distribution models of probability of occurrence, as available. For each VME indicator taxon we provide a brief review of their ecological function which will be important in future assessments of significant adverse impact on these habitats here and elsewhere.

Citation: Kenchington E, Murillo FJ, Lirette C, Sacau M, Koen-Alonso M, et al. (2014) Kernel Density Surface Modelling as a Means to Identify Significant Concentrations of Vulnerable Marine Ecosystem Indicators. PLoS ONE 9(10): e109365. doi:10.1371/journal.pone.0109365

Editor: Simon Thrush, University of Auckland, New Zealand

Received May 1, 2014; **Accepted** August 28, 2014; **Published** October 7, 2014

Copyright: © 2014 Kenchington et al. This is an open-access article distributed under the terms of the Creative Commons Attribution License, which permits unrestricted use, distribution, and reproduction in any medium, provided the original author and source are credited.

Data Availability: The authors confirm that all data underlying the findings are fully available without restriction. Data from Canadian research vessel surveys are deposited in the Ocean Biogeographic Information System (OBIS) at http://obiscanada.marinebiodiversity.ca/or http://www.iobis.org/. Spanish/EU data are available at Figshare with the DOI: http://dx.doi.org/10.6084/m9.figshare.1165479.

Funding: Funding was received from the International Governance Strategy (IGS) fund of the Department of Fisheries and Oceans to EK. The purpose of this fund is to strengthen scientific research in support of international governance of fisheries resources, support healthy ocean ecosystems, protect Canada's economic and environmental interests and position Canada as a global leader in high-seas issues. The funders had no role in study design, data collection and analysis, decision to publish, or preparation of the manuscript.

Competing Interests: The authors have declared that no competing interests exist.

* Email: Ellen.Kenchington@dfo-mpo.gc.ca

Introduction

The 2006 United Nations General Assembly Resolution 61/105 calls upon "States to take action immediately, individually and through regional fisheries management organizations and arrangements, and consistent with the precautionary approach and ecosystem approaches, to sustainably manage fish stocks and protect vulnerable marine ecosystems, including seamounts, hydrothermal vents and cold water corals, from destructive fishing practices, recognizing the immense importance and value of deep sea ecosystems and the biodiversity they contain".

The Food and Agriculture Organization (FAO) of the United Nations International Guidelines for the Management of Deep-sea Fisheries in the High Seas [1] provide general tools and considerations for the identification of vulnerable marine ecosystems (VMEs). They include a set of criteria that should be used, individually or in combination, for the identification process. Specifically:

"i. Uniqueness or rarity – an area or ecosystem that is unique or that contains rare species whose loss could not be compensated for by similar areas or ecosystems. These include:

- habitats that contain endemic species;
- habitats of rare, threatened or endangered species that occur only in discrete areas; or
- nurseries or discrete feeding, breeding, or spawning areas.

ii. Functional significance of the habitat – discrete areas or habitats that are necessary for the survival, function, spawning/reproduction or recovery of fish stocks, particular life history stages (e.g. nursery grounds or rearing areas), or of rare, threatened or endangered marine species.

iii. Fragility – an ecosystem that is highly susceptible to degradation by anthropogenic activities.

iv. Life-history traits of component species that make recovery difficult – ecosystems that are characterized by populations or assemblages of species with one or more of the following characteristics:

- slow growth rates;
- late age of maturity;
- low or unpredictable recruitment; or
- long-lived.

v. Structural complexity – an ecosystem that is characterized by complex physical structures created by significant concentrations of biotic and abiotic features. In these ecosystems, ecological processes are usually highly dependent on these structured systems. Further, such ecosystems often have high diversity, which is dependent on the structuring organisms." [1] The Northwest Atlantic Fisheries Organization (NAFO) is the regional fisheries management organization with regulatory competence over the fishing activities that take place in international waters in the northwest Atlantic. NAFO has reviewed the invertebrate catch from depth-stratified random research vessel trawl sets, undertaken in its regulatory area, against the FAO guidelines [2–5]. They identified large-sized sponges, large and small gorgonian corals, sea pens, erect bryozoans, sea squirts, crinoids (sea lilies) and cerianthid anemones as VME indicator species from a wider list of approximately 500 taxa residing in the area [4]. All qualified under the Structural Complexity criterion (see *v* above) in combination with their Fragility and Life-history traits (see *iii* and *iv* above respectively). The guidelines do not explicitly define the distinction between a VME and a VME indicator species/taxon, although it is clear that a single occurrence does not constitute a VME, nor does the full distribution of a species/taxon [1]. However, under the Structural Complexity criterion the term "significant concentration" is used to identify the level of aggregation which is expected, even though it is given without an operational context.

In landscape ecology, such hotspots are often detected with a spatially global threshold determined through comparison of the value for a given observation with locations in the neighbourhood of the observation, in order to incorporate an explicit consideration of space. We propose a novel method to identify "significant concentrations" of benthic structure-forming VME indicators using a global threshold determined from geospatial analyses. Our approach serves to objectively separate dense aggregations that form habitats from the broader distribution of the species in question, and so serves to distinguish VMEs proper (i.e., structurally complex habitats) from single/low frequency occurrences of VME indicator species. We apply our method to four of the highly aggregating structure-forming megafaunal groups identified as VME indicator species/taxa by NAFO - specifically to large-sized sponges, sea pens, and small and large gorgonian corals. Our approach does not address questions of impairment of ecological function which ultimately are important in assessing significant adverse impacts on these habitats [1], nor does it attempt to predict biomass or occurrence in unsampled areas. It is particularly relevant to the analyses of VME-indicator catch from research vessel survey data series on continental shelves and slopes. Modeled outputs are ground-truthed using data reported from independent surveys and published species distribution analyses predicting the probability of occurrence of the VME indicator taxa based on a suite of environmental variables.

Materials and Methods

Study Area

The high seas study area in the northwest Atlantic is bounded by the Canadian exclusive economic zone (EEZ) in the west and by the 2500 m depth contour along the continental slope. This includes the Nose and Tail of Grand Bank, east of Newfoundland, Canada, and the Flemish Cap, a plateau of roughly 200 km radius at the 500 m isobath and separated from Grand Bank by the Flemish Pass which reaches depths of 1200 m (Figure 1). The study area falls within the 3LMNO management Divisions of NAFO and is referred to as the NAFO Regulatory Area (NRA).

Data

The study area is typically sampled on an annual basis by research vessel (RV) bottom trawl surveys carried out for the assessment of fish stocks by Canada and the European Union (Spain). This study did not involve human participants, specimens or tissue samples. All of the animals in this study are invertebrates and so do not require an ethics statement. Fish were caught on the same surveys but did not form a part of this study. All scientific field surveys conducted in the NAFO Regulatory area are approved by the NAFO Scientific Council on an annual basis. All of the surveys carried out for this study followed this procedure. For future surveys applicants should contact the Chair of the NAFO Scientific Council. The name and contact information of the Chair is available on the NAFO Website: http://www.nafo.int/.

All surveys follow a depth-stratified random sampling design to 1500 m optimized for the target species with vessel speeds of approximately 3 knots, however there are differences in the type and size of the gears used and the duration of the trawl sets (Table 1).

Collectively these surveys provided 2593 geo-referenced records of sponge biomass and 1478 records of coral biomass distributed as illustrated in Figure 2. Each of these records was grouped into one of the broader VME indicator species/taxa: large-size sponges, sea pens, small gorgonian corals or large gorgonian corals. Sponges were not consistently identified to species level in the surveys, however all of the large catches were comprised of massive sponges of the Families Geodiidae and Ancorinidae [6]. Sea pen catches (Cnidarian Order Pennatulacea) were dominated by two whip-like species *Anthoptilum grandiflorum* and *Halipteris finmarchica*, and the smaller fleshy *Pennatula aculeata* [7,8] though 11 species have been identified [9]. Small gorgonians coral catches (Cnidarian Order Alcyonacea) were predominately of the species *Acanella arbuscula*, although other species with similar environmental requirements, such as *Radicipes gracilis* (Cnidarian Order Alcyonacea) were included; the large gorgonian corals (Cnidarian Order Alcyonacea) comprised *Acanthogorgia* spp., *Keratoisis* spp., *Paragorgia arborea, P. johnsoni, Paramuricea* spp., and *Primnoa resedaeformis* [9].

Data Treatment

The data were drawn from three different combinations of gear type and trawling duration (Table 1). All catch weight distributions were highly right-skewed due to large numbers of small catches with few very large catches (Figure 3) as is characteristic of highly aggregating species. When spatially visualized the large

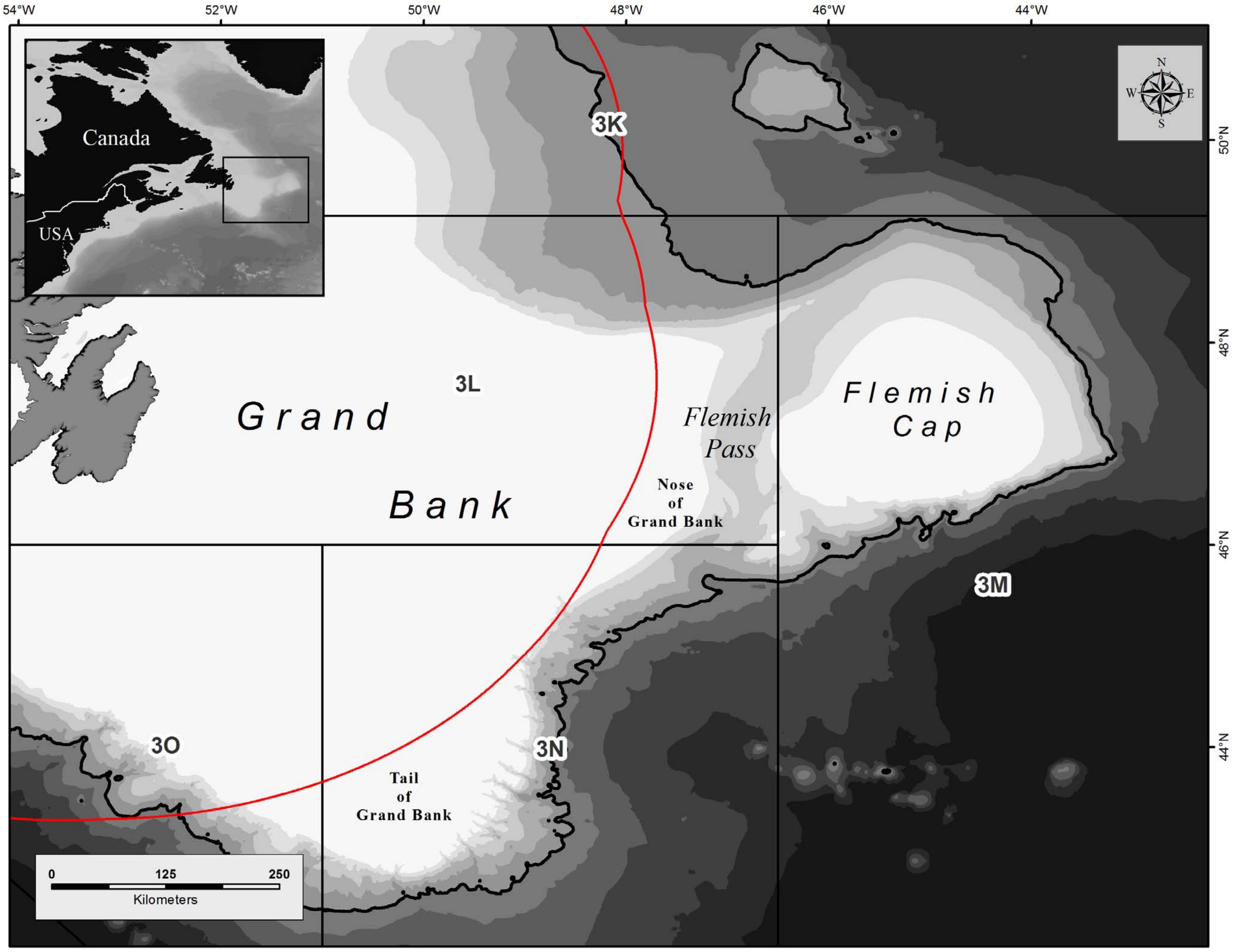

Figure 1. Location of the study area in the international waters east of Newfoundland and Labrador, Canada. Red line indicates the Canadian Exclusive Economic Zone. Heavy black line indicates the 2500 m depth contour. NAFO management divisions are indicated.
doi:10.1371/journal.pone.0109365.g001

catches typically co-occurred in close proximity, whereas the smaller catches were broadly distributed.

To assess whether the different survey data should be used separately or in combination for each VME indicator taxon, we applied non-parametric statistics to the catch biomass from each of the three gear/duration data sets using all of the biomass data and only those data above arbitrary weight classes. Because each of the surveys covered different and largely non-overlapping spatial extent (Figure 2), natural differences in biomass are confounded in this approach and could enhance or mask differences between

Table 1. Details of research vessel survey data of coral and sponge biomass for the study area.

Programme	Period	NAFO Division	Trawl gear type	Mesh size in codend liner (mm)	Trawl duration (min)	Average wingspread (m)
Spanish 3NO Survey (IEO)	2002–2013	3NO	Campelen 1800	20	30	24.2–31.9
EU Flemish Cap Survey (IEO, IIM, IPIMAR)	2003–2013	3M	Lofoten	35	30	13.89
Spanish 3L Survey (IEO)	2003–2013	3L	Campelen 1800	20	30	24.2–31.9
DFO NL Multi-species Surveys (DFO)	1995–2012	3LNO	Campelen 1800	12.7	15	15–20

Notes: EU, European Union; DFO, Department of Fisheries and Oceans; NL, Newfoundland and Labrador; IEO, Instituto Español de Oceanografia; IIM, Instituto de Investigaciones Marinas; IPMA, Instituto Português do Mar e da Atmosfera.
doi:10.1371/journal.pone.0109365.t001

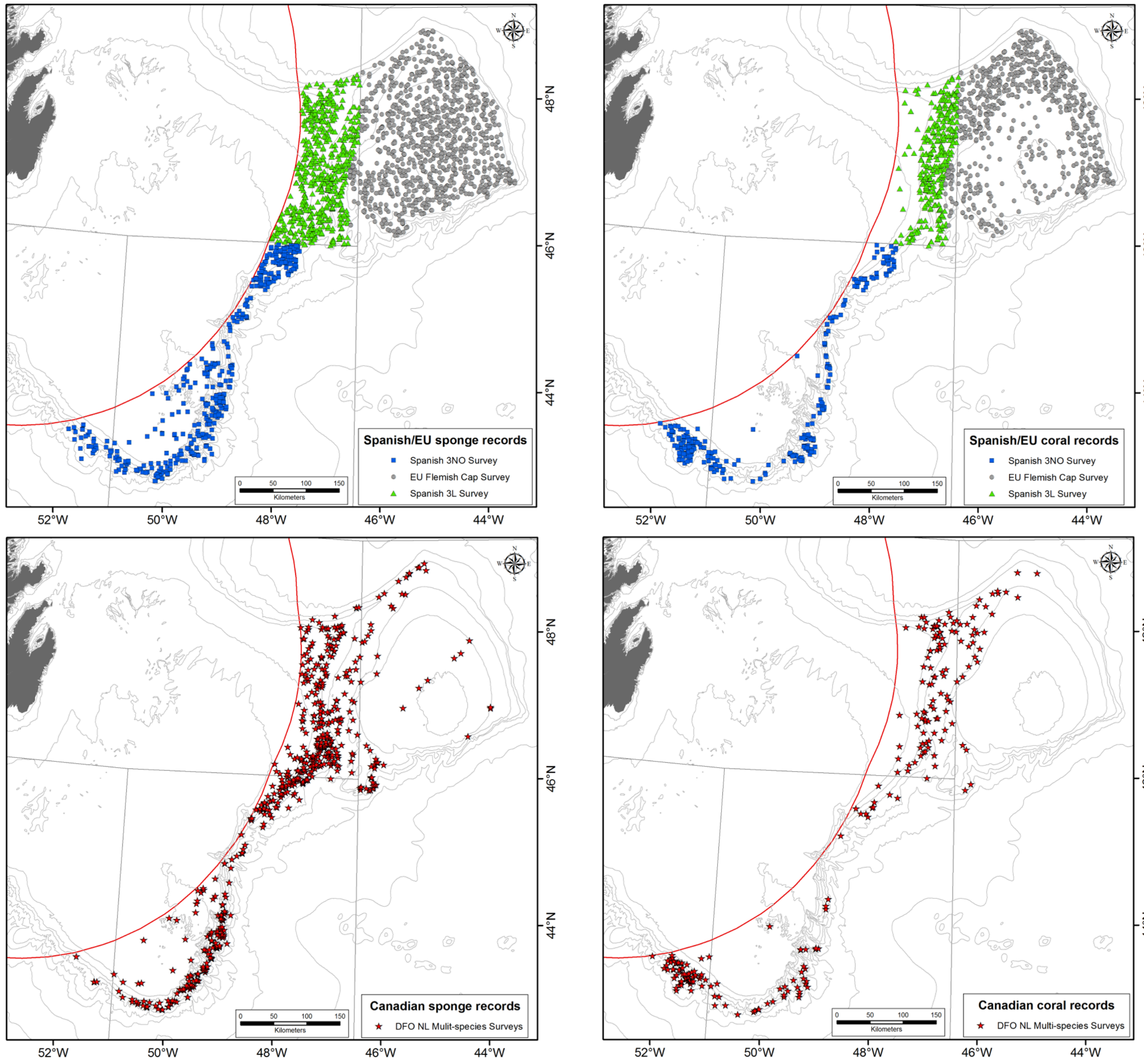

Figure 2. Location of trawl sets for the research vessel surveys in the NAFO Regulatory Area (Divs. 3LMNO) used for analyses (Table 1). Left panels: Sponges; Right Panels: Corals.
doi:10.1371/journal.pone.0109365.g002

gears and/or trawl duration. This effect is most likely to affect tests involving gear comparison as the Lofoten gear was used almost exclusively on the Flemish Cap while the Campelen trawls were deployed in the Flemish Pass and on Grand Bank. Comparison of trawl duration effects between the surveys using Campelen gear cover a similar spatial extent and so are less likely to be confounded by geospatial differences in catchability (Figure 2).

Two-sample Kolmogorov-Smirnov tests using only data collected with the Campelen gear were performed to evaluate the influence of trawl duration (15 min *vs.* 30 min), a proxy of trawl length. When no significant differences were found, the data sets were combined and tested against the Lofoten gear data, to evaluate the influence of the gear type. Further, graphs of trawl length, calculated from vessel speed and duration, vs. VME indicator taxon catch weight, and of the cumulative frequency distributions were used to visualize these data.

Kernel Density Estimation and Selection of Aggregation Thresholds

The non-parametric kernel density algorithm has been increasingly used in ecology to identify home ranges from tagging data [10–12] and for identifying hotspots within a landscape [13,14] using a neighbour-based approach. For the later application, ecological hotspots (that is areas of high abundance or biomass), are defined relative to the spatial distribution of the data and are detected through the application of a spatially-defined threshold [13]. We used kernel density estimation (KDE) to create a modelled biomass surface for each VME indicator taxon using the start positions of each RV trawl set. A quadratic (Epanechnikov) kernel density function was used to fit a smooth curve over each data point in ArcGIS 10 (ESRI Canada Limited, Toronto, Ontario) using the UTM projected coordinate system North

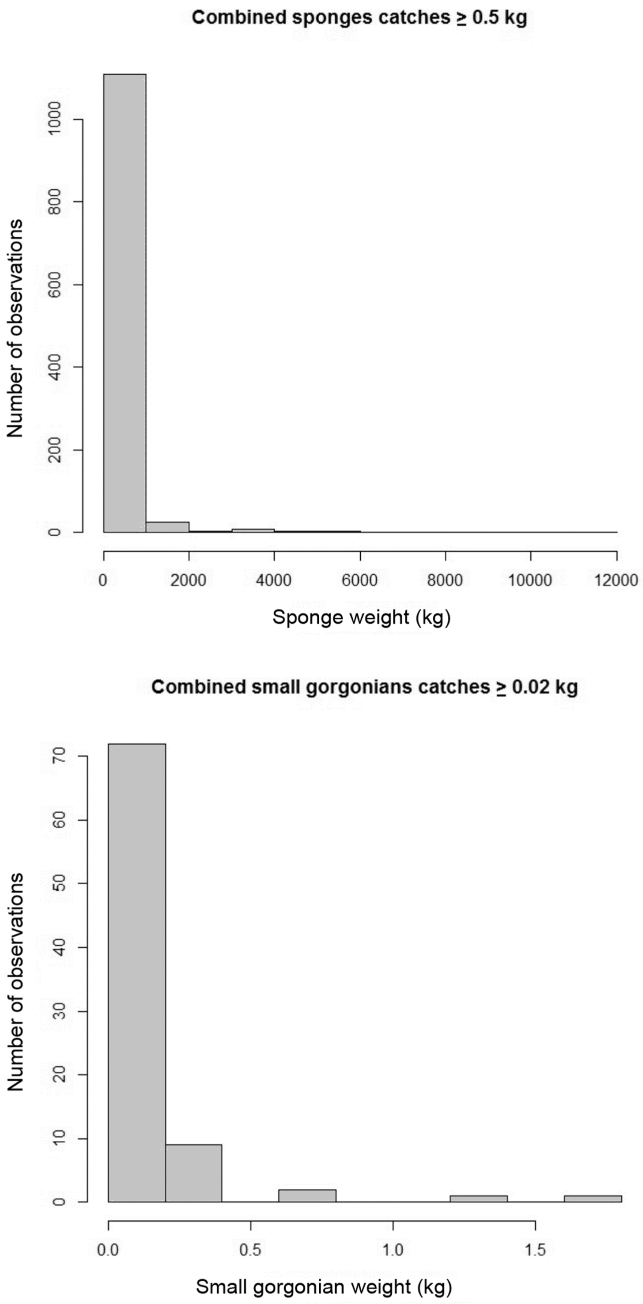

Figure 3. Catch weight distribution for all non-zero data of sponges (upper left), sea pens (upper right), small gorgonian corals (lower left) and large gorgonian corals (lower right).
doi:10.1371/journal.pone.0109365.g003

American Datum 1983 Zone 23. We used an optimum search radius based on the tool's default calculation which is the shortest of the width or height of the output extent in the output spatial reference, divided by 30. In all cases the width was the shortest extent and the search radii were 22.6 km for sponges and sea pens, 22.1 km for small gorgonian corals and 16.6 km for large gorgonian corals. The surface value is highest at the location of the point and decreases outwards in all directions to reach zero at the search radius distance to define a circular neighbourhood for each point observation. Output cell size (resolution) was also based on the tool's default which is the shorter of the width or height of the output extent, divided by 250. Again using the width of the spatial extent the output cell size was ~2.7 km for sponges, sea pens and small gorgonian corals and 2.0 km for large gorgonian corals. The density at each output raster cell was calculated by adding the values of all the kernel surfaces within each cell.

Once the kernel density surface was produced, it was used to estimate the area covered by the original data points at or above selected biomass threshold levels along the kernel contours. This calculation was repeated for successively decreasing biomass threshold values in order to generate threshold-area curves; these curves allowed for identification of threshold levels associated with natural changes in the pattern of spatial aggregation in the data [7,8,15]. The polygons determining that threshold were then

superimposed over the original kernel surface to differentiate the hot and non-hot spots.

The structure-forming VME indicators examined here are all aggregating taxa forming habitats commonly referred to as sponge grounds, sea pen fields and coral forests. Consequently we expected to see biomass hotspots appearing as tightly defined spatial units in the overall landscape with sharp discontinuities between neighbouring hotpots.

Typically, for these VME indicator species/taxa that form habitats through dense aggregations, the threshold-area curves initially show a slow increase in total area as the threshold values decrease. This slow increase in area reflects the fact that the arbitrary thresholds keep "mapping out" the areas that contain the dense aggregations of VME indicator species/taxa (i.e., better delineating the areas of high density, where density may decrease near their boundaries, while also by starting to incorporate smaller new aggregation areas with relatively lower densities). After this initial "phase" of slow increase in area, the threshold-area curves show a rapid and sharp increase in area as the thresholds keep decreasing; this rapid increase in area is associated with thresholds values that are beginning to capture isolated/non-aggregating individuals of the VME indicator species/taxa. Finally, as the thresholds reach its lowest value, the area covered often stabilizes again, reflecting the entire distribution of the VME indicator species/taxa in the study area.

Identification of biomass thresholds representing "significant concentrations" of VME indicator species/taxa correspond to thresholds demarcating the phase of rapid increase in area. When interpreting the catch weight defining the significant concentrations a number of criteria are simultaneously considered: 1) identification of the catch biomass which show the largest change in area after the initial establishment of the aggregations; 2) consideration of the number of data points contributing to those changes in area between successive catch thresholds; 3) examination of the spatial relationship of the polygons created by biomass thresholds greater and lesser than the potential threshold using GIS, and 4) the position of the new data points relative to previously established polygons. These two last criteria are the spatial component to criterion 2 and are necessary as polygon area can increase by the joining of two or more high density polygons. If this occurs the evidence for connecting the areas (i.e., number of points between the smaller areas) is reviewed. In this instance the threshold is considered to be valid when there is an increase in area through a reasonable number of widely spaced data points. Cases for rejecting the threshold other than insufficient data include: 1) joining of smaller polygons with little evidence for a continuous distribution within the newly formed area; 2) a gradual increase in area with every new polygon added, creating a situation where no one successive change in area is especially larger or smaller than others (this indicates that there is no aggregation); 3) an increase in area established by creation of new areas of very low density; and 4) no large increase in area.

Independent Assessments of Modelled Outputs from Published Sources

Independent data, useful for validating the modelled results, have been collected through NEREIDA (http://www.nafo.int/science/frames/nereida), a multidisciplinary research programme operating within the study area. Data from NEREIDA rock dredges, box corers and underwater imagery [16] offer an opportunity to validate modelled results. In particular, data from nine *in situ* benthic imagery transects conducted in the Sackville Spur and western Flemish Cap slope/Flemish Pass areas in 2009 and 2010 have been processed [17]. These were primarily in areas predicted to have significant concentrations of sponges identified using the above approach (i.e., sponge grounds) [16–18] and were complemented by collection of box core samples [19].

Species distribution models (SDMs) use environmental data to predict the probability of occurrence of a species or habitat. SDMs using random forest models have been applied to the presence/absence of sponge grounds, sea pens and large gorgonian corals in the study area [20,21]. These drew on the same response data sources used in our analyses and so are not fully independent; however they have, through their use of the environmental predictors, capacity to extrapolate between point observations in unsampled areas or where data are sparse.

Results

Analyses of the catch weight distributions between trawl durations (length) and gear types indicated that catch weights of sponges $\geq$0.5 kg, of sea pens $\geq$0.02 kg, of small gorgonian corals $\geq$0.1 kg and of large gorgonian corals $\geq$0.1 kg did not differ significantly between trawl duration of 15 min and of 30 min (Table 2). This is reinforced by the scatterplots presented in Figure 4 that show that above those thresholds, there is no relationship between the catch weight and length of tow. This is consistent with the aggregated distribution of the VME indicator taxa and the need for alignment between the trawl path and the maximum dimension of the aggregation to create a linear relationship. Further, except for the small gorgonian corals, the Canadian records are all above 0.01 kg catch, whereas Spanish/EU data record values to 0.001 kg; this could be due to different sampling protocols or scale precision. All of these weight thresholds for combining the data represent only a few individuals or less of each VME indicator taxon at their lowest values.

With the exception of the sea pens, none of the catch weights of the VME indicator taxa differed between gear type (Campelen and Lofoten) at the thresholds established for combining the data based on trawl duration (Table 2), justifying combining all of the data above those catch weights for further analyses. Sea pen catches $\geq$0.2 kg showed no significant difference between the types of gear used (Table 2), and so data records less than 0.2 kg were discarded.

For the small gorgonian corals the data reduction incurred through the above analyses (combination of data $\geq$0.1 kg catch weight), drastically reduced the number of point observations available for KDE analysis to less than 10% of the total small gorgonians records ($N = 36$; Table 2). In order to maximize the use of the data while respecting the differences in catch weight distribution amongst the surveys, we ran two separate KDE analyses: one model for the Flemish Cap collected with Lofoten gear ($N = 145$) and another for a subset of the spatial extent surveyed with the Campelen gear on the Tail of Grand Bank (3NO Divs.) combining survey catches for that area that were $\geq$ 0.02 kg ($N = 85$; $KS = 0.289$; $p = 0.058$). Similar concerns over the number of point observations for large gorgonian corals could not be addressed through this approach; however that taxon had a smaller overall spatial extent of observations.

Sponges

A KDE surface of sponge biomass was modelled from the 1154 sponge records $\geq$0. 5 kg (Figure 5) and the areas under polygons of decreasing weight thresholds were calculated. Between 75 kg and 50 kg polygon area increased 1.25 times from 22,439 km^2 to 28,112 km^2 through the addition of 27 new points, 13 of them in new areas outside of the polygons defined by the 75 kg threshold. Consequently, the threshold value of 75 kg meets the assessment

Table 2. Kolmogorov-Smirnov (KS) two-sample tests of the similarity of the catch weight distribution for each VME taxon testing for effects of trawl duration (15 min vs. 30 min) and gear type (Campelen vs. Lofoten trawls) (see Table 1).

VME Indicator Taxon	Comparison Groups (Samples)	Number of Records (N) for Each Test Sample for a Given Catch Biomass Threshold	KS	P
Sponges	Campelen 15 min trawl *vs.* Campelen 30 min trawl	>0 kg ($N_{Canada}=553$, $N_{EU\text{-}Spain}=1024$)	0.288	<0.001*
		≥0. 1 kg ($N_{Canada}=491$, $N_{EU\text{-}Spain}=640$)	0.142	<0.001*
		≥0. 5 kg ($N_{Canada}=391$, $N_{EU\text{-}Spain}=439$)	0.069	0.279
		≥1 kg ($N_{Canada}=339$, $N_{EU\text{-}Spain}=354$)	0.055	0.683
		≥10 kg ($N_{Canada}=137$, $N_{EU\text{-}Spain}=136$)	0.131	0.193
	Combined Campelen trawls *vs.* Lofoten trawl	≥0. 5 kg ($N_{Campelen}=830$, $N_{Lofoten}=324$)	0.065	0.284
		≥1 kg ($N_{Campelen}=693$, $N_{Lofoten}=255$)	0.059	0.5443
		≥10 kg ($N_{Campelen}=273$, $N_{Lofoten}=97$)	0.137	0.138
Sea Pens	Campelen 15 min trawl *vs.* Campelen 30 min trawl	>0 kg ($N_{Canada}=183$, $N_{EU\text{-}Spain}=489$)	0.279	<0.001*
		≥0. 01 kg ($N_{Canada}=182$, $N_{EU\text{-}Spain}=350$)	0.172	0.002*
		≥0.02 kg ($N_{Canada}=146$, $N_{EU\text{-}Spain}=299$)	0.099	0.288
		≥0.05 kg ($N_{Canada}=105$, $N_{EU\text{-}Spain}=196$)	0.094	0.583
		≥0.1 kg ($N_{Canada}=72$, $N_{EU\text{-}Spain}=118$)	0.119	0.550
		≥0.2 kg ($N_{Canada}=35$, $N_{EU\text{-}Spain}=61$)	0.207	0.299
	Combined Campelen trawls *vs.* Lofoten trawl	≥0.02 kg ($N_{Campelen}=445$, $N_{Lofoten}=436$)	0.170	<0.001*
		≥0.05 kg ($N_{Campelen}=301$, $N_{Lofoten}=324$)	0.199	<0.001*
		≥0.1 kg ($N_{Campelen}=190$, $N_{Lofoten}=239$)	0.218	<0.001*
		≥0.2 kg ($N_{Campelen}=96$, $N_{Lofoten}=166$)	0.160	0.087
		≥0.5 kg ($N_{Campelen}=32$, $N_{Lofoten}=71$)	0.134	0.822
Small Gorgonian Corals	Campelen 15 min trawl *vs.* Campelen 30 min trawl	>0 kg ($N_{Canada}=87$, $N_{EU\text{-}Spain}=172$)	0.483	<0.001*
		≥0. 01 kg ($N_{Canada}=83$, $N_{EU\text{-}Spain}=81$)	0.360	<0.001*
		≥0.02 kg ($N_{Canada}=47$, $N_{EU\text{-}Spain}=55$)	0.271	0.049*
		≥0.05 kg ($N_{Canada}=27$, $N_{EU\text{-}Spain}=25$)	0.430	0.017*
		≥0.1 kg ($N_{Canada}=19$, $N_{EU\text{-}Spain}=10$)	0.395	0.259
	Combined Campelen trawls *vs.* Lofoten trawl	≥0.1 kg ($N_{Campelen}=29$, $N_{Lofoten}=7$)	0.374	0.408
Large Gorgonian Corals	Campelen 15 min trawl *vs.* Campelen 30 min trawl	>0 kg ($N_{Canada}=42$, $N_{EU\text{-}Spain}=75$)	0.413	<0.001*
		≥0. 01 kg ($N_{Canada}=42$, $N_{EU\text{-}Spain}=44$)	0.352	0.010*
		≥0.02 kg ($N_{Canada}=29$, $N_{EU\text{-}Spain}=38$)	0.331	0.054
		≥0.05 kg ($N_{Canada}=21$, $N_{EU\text{-}Spain}=28$)	0.441	0.019*
		≥0.1 kg ($N_{Canada}=13$, $N_{EU\text{-}Spain}=27$)	0.171	0.960
	Combined Campelen trawls *vs.* Lofoten trawl	≥0.1 kg ($N_{Campelen}=40$, $N_{Lofoten}=18$)	0.292	0.242

Asterisks indicate significant differences at $\alpha=0.05$.
doi:10.1371/journal.pone.0109365.t002

criteria and can be used to approximate the limits of the sponge grounds (that is, significant concentrations of sponges, equating that density to a VME proper) (Figure 6). The 75 kg polygon area encloses 97.87% of the total sponge biomass recorded from this area and represents 19.73% of the sampled area, confirming high concentration of sponge biomass.

In situ benthic camera surveys and box core sampling have supported the occurrence of significant concentrations of sponges in Flemish Pass [18] and on Sackville Spur [16,17,19] (Figure 7) as well as on the northeast slope of Flemish Cap [17]. SDMs have recently been applied to predict the probability of occurrence of sponge grounds in the study area [20] and show excellent concordance with the location of the significant concentrations

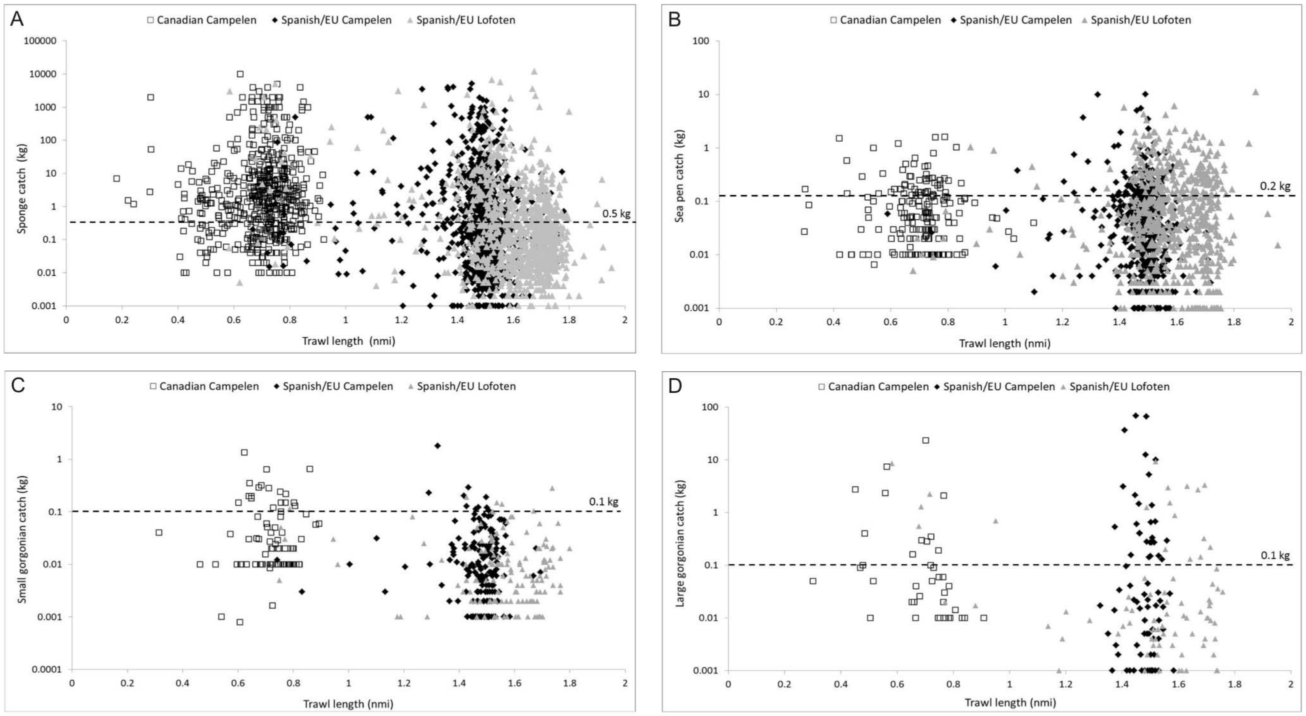

Figure 4. Catch weight (kg) of each VME indicator taxon in relation of trawl length (nmi) for each of the surveys indicated in Table 1 (Canadian surveys with Campelen gear, Spanish/EU surveys with Campelen gear and Spanish/EU surveys with Lofoten gear). The dashed line indicates the catch value above which the distributions are not significantly different (Table 2). Note that the y axis is in logarithmic scale. A) Sponges. B) Sea pens. C) Small gorgonian corals. D) Large gorgonian corals.
doi:10.1371/journal.pone.0109365.g004

identified through our approach. Sponge grounds in the study area were primarily observed and predicted to exist in areas with high (>0.1 m/s) maximum bottom current [20].

Sea Pens

A KDE surface of sea pen biomass was modelled from 261 sea pen records ≥0.2 kg (Figure 8). Similar to the sponges, the area occupied by the highest catches is relatively constant, after an

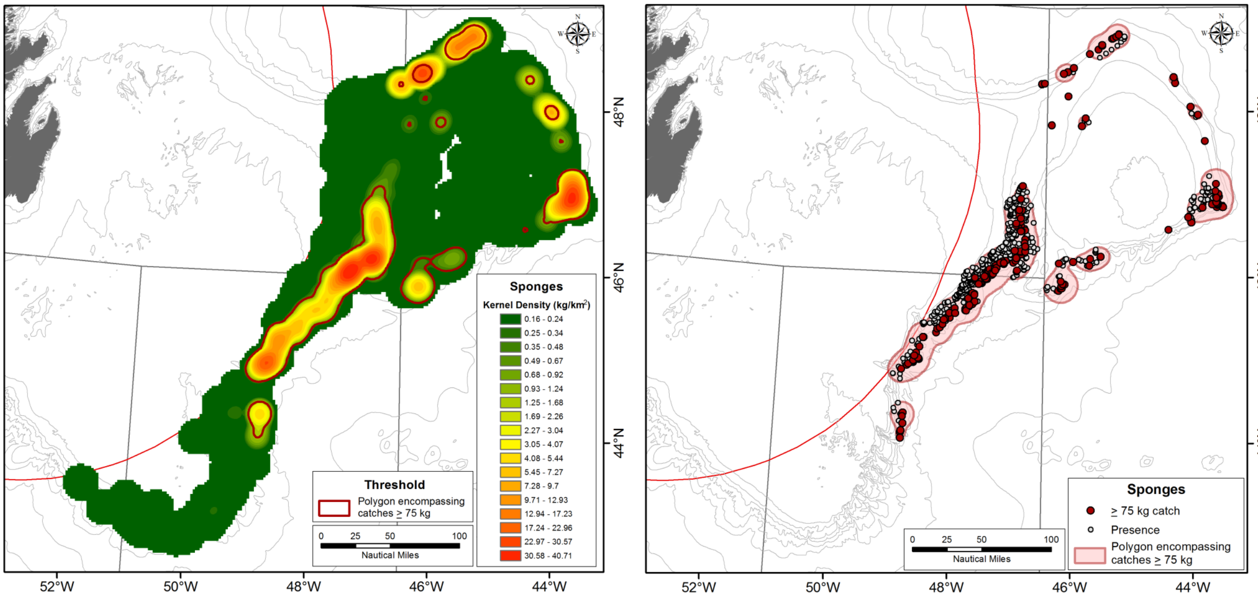

Figure 5. Left panel: Kernel density distribution of sponges in the NAFO Regulatory area with the 75 kg density polygons defining significant concentrations determined from aerial expansion thresholds superimposed in red. The green areas represent low sponge densities while the red areas indicate high sponge densities. Right panel: The location of catches greater than 75 kg (red circle) and smaller sponge catches (open circles) within the 75 kg density polygons defining the sponge ground VMEs.
doi:10.1371/journal.pone.0109365.g005

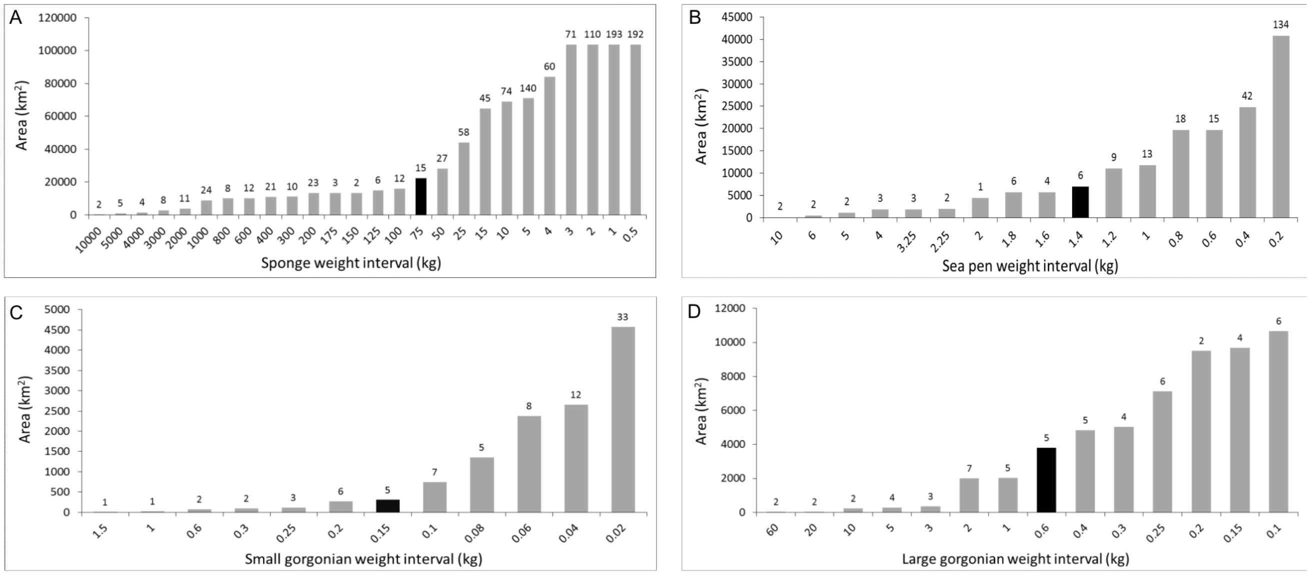

Figure 6. Histograms of the area occupied by successive weight thresholds of VME Indicator taxa. The numbers of additional point observations (over the preceding higher weight threshold) used to create each polygon are indicated above the each bar. A) Sponges. B) Sea pens. C) Small gorgonian corals. D) Large gorgonian corals.
doi:10.1371/journal.pone.0109365.g006

initial increase, until the 2.25 kg interval. The area shows a first increase between 2.25 and 2 kg intervals, but this is created through the inclusion of a datum. After this, the area occupied is relatively constant until 1.4 kg. Between the 1.4 and 1.2 kg intervals the polygon area increases 1.58 times from 6,983 km^2 to 11,050 km^2 and meets the criteria outlined above for identification of a "significant concentration" threshold. The 1.4 kg polygon area encloses the 59.28% of the total sea pen biomass recorded and this area represents 5.10% of the sampled area (Figure 6).

Rock dredge data collected under the NEREIDA programme independently confirmed the presence of sea pens in the areas of significant concentrations identified above. There were no underwater imagery stations coincident with the significant concentrations of sea pens in the NRA, however, congruence between *in situ* observations of sea pen fields and locations of high research vessel trawl catches has been observed in the nearby Laurentian Channel [8] (Figure 7). The SDMs produced for sea pens in the study area show agreement with the kernel density surface. Both highlight a horse-shoe shaped distribution on Flemish Cap and significant concentrations and areas of high probability of occurrence on the Tail of Grand Bank, particularly adjacent to the Canadian EEZ in NAFO Div. 3O [21].

Small Gorgonian Corals

Two KDE surfaces for small gorgonian coral biomass were produced, one for the Flemish Cap and one for the Tail of Grand Bank as described above. For the Flemish Cap, the majority of the records were small catches (≤0.01 kg) with only 1 catch greater than 0.2 kg and 6 greater than 0.1 kg. These small catches were not highly aggregated and the analyses of the area occupied by successive density polygons supported that observation, as no clear threshold emerged with sufficient support following the criteria outlined above for identification of significant concentrations.

For the Tail of Grand Bank, the assessment criteria applied to the KDE surface identified 0.15 kg as the threshold for defining significant concentrations of small gorgonian corals (Figure 6). When superimposed on the KDE surface (Figure 9), the 0.15 kg density polygon captures all of the highest density areas (red colour on Figure 9) from the kernel analysis. Review of the data surrounding these polygons showed that three of the areas (marked with arrows on Figure 9) are based on single records with null records surrounding them. These areas require further data to determine the spatial extent of the concentrations.

Although small, the highly-branched nature of small gorgonian corals such as *Acanella arbuscula* (Figure 7), and their ability to form small but dense aggregations deems them habitat-forming, especially in areas of low topographical relief [22]. Rock dredge samples collected through the NEREIDA programme for the most part were not co-located with areas of significant concentrations of small gorgonian corals except for two locations on the Tail of Grand Bank. Both of those samples contained small gorgonian corals with high relative abundance compared to other rock dredge samples with small gorgonian coral presence. SDMs have not yet been published for this taxon.

Large Gorgonian Corals

Large gorgonian corals found in the study area (Figure 7) are very fragile and their representation in the catch is most often in the form of coral fragments rather than whole colonies. The KDE distribution identified large gorgonian coral catches in Flemish Pass, on Beothuk Knoll and on the southeastern corner of Flemish Cap (Figure 10). The 0.6 kg/RV tow density threshold emerged as defining significant concentrations (Figure 6) and when superimposed on the kernel density surface (Figure 10), it can be seen that all of the highest density areas (red colour on Figure 10) from the kernel analysis and other smaller catches are found within the defining polygons. As for the small gorgonian corals, four of the areas are based on single records. Of these four, the catch closest to the area of highest densities in the western section of the Flemish Cap, at around 500 m depth, is surrounded by other large gorgonian catches below the threshold and likely represents a significant concentration, especially considering the

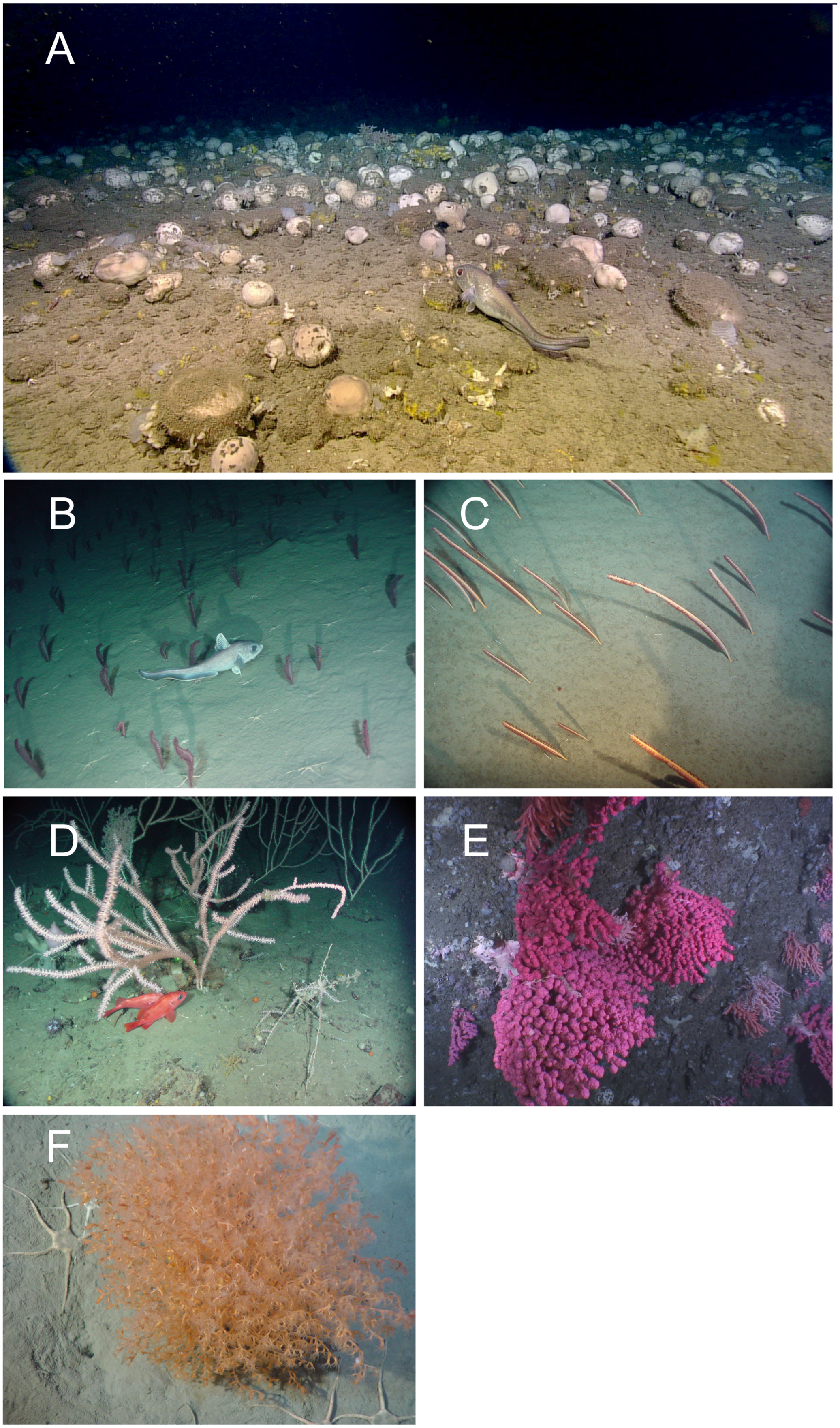
A
B
C
D
E
F

Figure 7. Underwater images were taken from the locations of large research vessel trawl catches of (A) Geodid-dominated sponge grounds from the northeast Flemish Cap in the NAFO Regulatory area (NRA), (B) sea pen fields in the Laurentian Channel south of the NRA dominated by species of *Pennatula*, a smaller flesher sea pen, (C) sea pen fields in the Laurentian Channel south of the NRA dominated by the taller whip-like species *Halipteris finmarchica*, (D) large gorgonian corals in the Laurentian Channel south of the NRA (*Keratoisis* sp.), (E) large gorgonian corals from the southern wall of Flemish Pass in the NRA (*Paragorgia* sp.), (F) *Acanella arbuscula* from the Gully Marine Protected Area on the Scotian Shelf showing its dense branching architecture.
doi:10.1371/journal.pone.0109365.g007

fragility of these taxa. However, the other three areas identified as significant concentrations (north Flemish Pass and slope of the Tail of the Grand Bank) are surrounded with null records and more data should be collected to resolve the spatial extent of those aggregations.

The SDM [21] predicts large gorgonian corals to be present with high probability in the area of significant concentrations of large gorgonian corals in Flemish Pass identified using the kernel density analyses. However, the area of highest probability of occurrence is along the deep, south and eastern slopes of Flemish Cap where there is little data available from the surveys. The SDM also predicts that large gorgonian corals will be present in the deeper water of the Sackville Spur area. Although underwater cameras have confirmed the presence of large gorgonian corals in the eastern portion of that area (Figure 7) they did not appear in the western transects [17]. Although the habitat may be suitable on Sackville Spur the dense sponge grounds in this area may out compete the coral.

Discussion

This study has demonstrated that kernel density analysis is useful for identifying "significant concentrations" [*sensu* 1] or hotspots from point observations of VME indicator taxon biomass from research vessel survey catches. Where independent data sources could be used to validate the presence of VMEs in the areas so identified, they consistently confirmed the modelled results.

We chose a kernel density estimation approach, over interpolation, to identify the location of biomass hotspots from a known distribution, as this method enhances habitat edges or boundaries [13] and is non-parametric. Density estimation counts the number of discrete objects in a given area and creates a field from those objects, whereas interpolation estimates the data values for points between the data. Geostatistical interpolation methods of producing biomass surfaces (e.g., kriging) require continuous and normally distributed data, while exact deterministic interpolation methods (e.g., inverse distance weighted (IDW)) dramatically underestimate areas of high biomass in right-skewed data. The highly aggregated nature of the VME indicator taxa results in a large number of zero catches and high variability in the positive catches, making it difficult to model an appropriate error term with alternative methods for producing biomass surfaces such as GLM/GAM which can give both prediction and uncertainty surfaces. Where such models have been applied to research vessel trawl-caught abundance of corals and sponges in the Pacific northwest [23], the best model fits explained between 20 and 25% of the deviance and produced low R^2 (0.04–0.21). Interestingly, in that study, the location variable (latitude, longitude) was significant in all models and accounted for ∼50% of the explanatory power of most models; removing that term eroded model performance whilst including it prevented extrapolation outside of the spatial extent of the data. This dependence on location likely arises from spatial autocorrelation due to the high degree of aggregation in these species and diminishes the value that such models might have over simpler density estimation approaches. Simple plotting

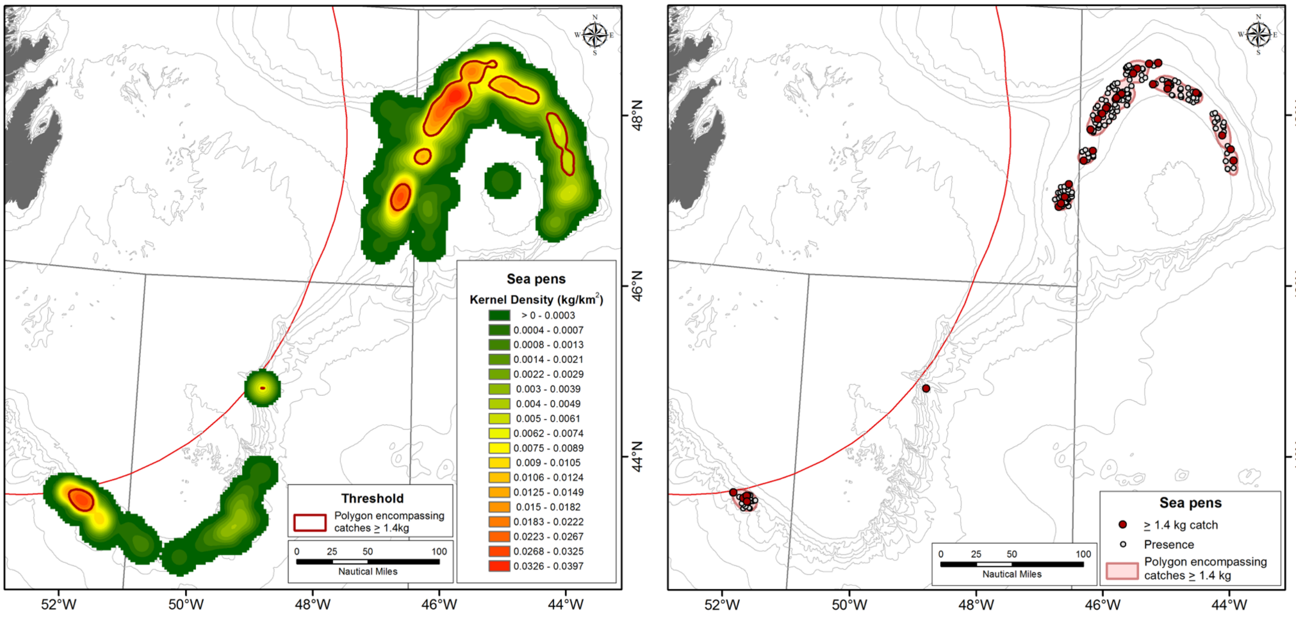

Figure 8. Left panel: Kernel density distribution of sea pens in the NAFO Regulatory area with the 1.4 kg density polygons defining the sea pen field VMEs superimposed in red. The green areas represent low sea pen densities while the red areas indicate high sea pen densities. Right panel: The location of catches greater than 1.4 kg (red circle) and smaller sea pen catches (open circles) within the 1.4 kg density polygons defining the sea pen field VMEs.
doi:10.1371/journal.pone.0109365.g008

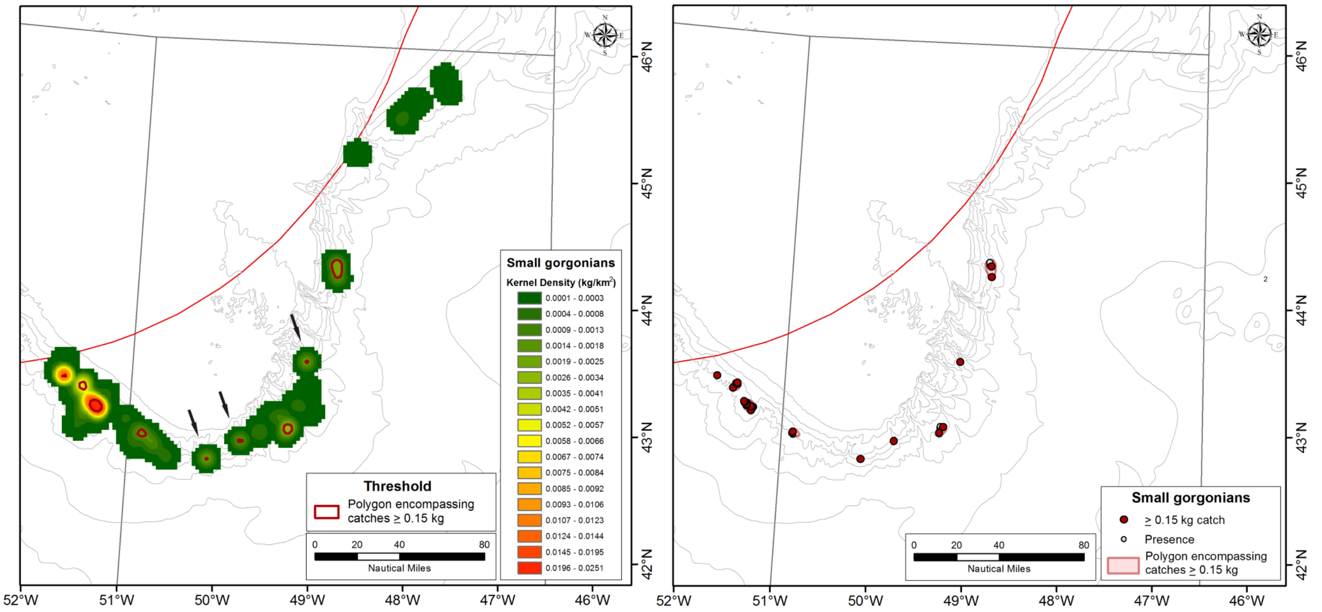

Figure 9. Left panel: Kernel density distribution of small gorgonian corals (primarily *Acanella arbuscula*) on the Tail of Grand Bank in the NAFO Regulatory area with the 0.15 kg density polygons superimposed in red. Arrows point to catches that appear to be isolated and require more data to establish their spatial extent. Right panel: The location of catches greater than 0.15 kg (red circle) and smaller small gorgonian coral catches (open circles) within the 0.15 kg density polygons defining the small gorgonian coral VMEs.
doi:10.1371/journal.pone.0109365.g009

of standardized biomass point data would allow for visualizing areas of dense aggregation but any delineation of a global threshold to separate "significant concentrations" would be necessarily subjective.

The production of a smoothed biomass surface is necessary for application of our method for determining a spatially global threshold to differentiate hot and non-hot locations. Our novel use of the change in area of the polygons encircling catches of a given magnitude allows for the area of aggregation (i.e., the significant concentration or VME) to be distinguished from the broader low density distribution of the taxon. We are unaware of any other approach to arrive at a threshold density that deals with this issue from a biological basis other than the use of the point of maximum curvature [13]. However that approach is not necessarily linked to

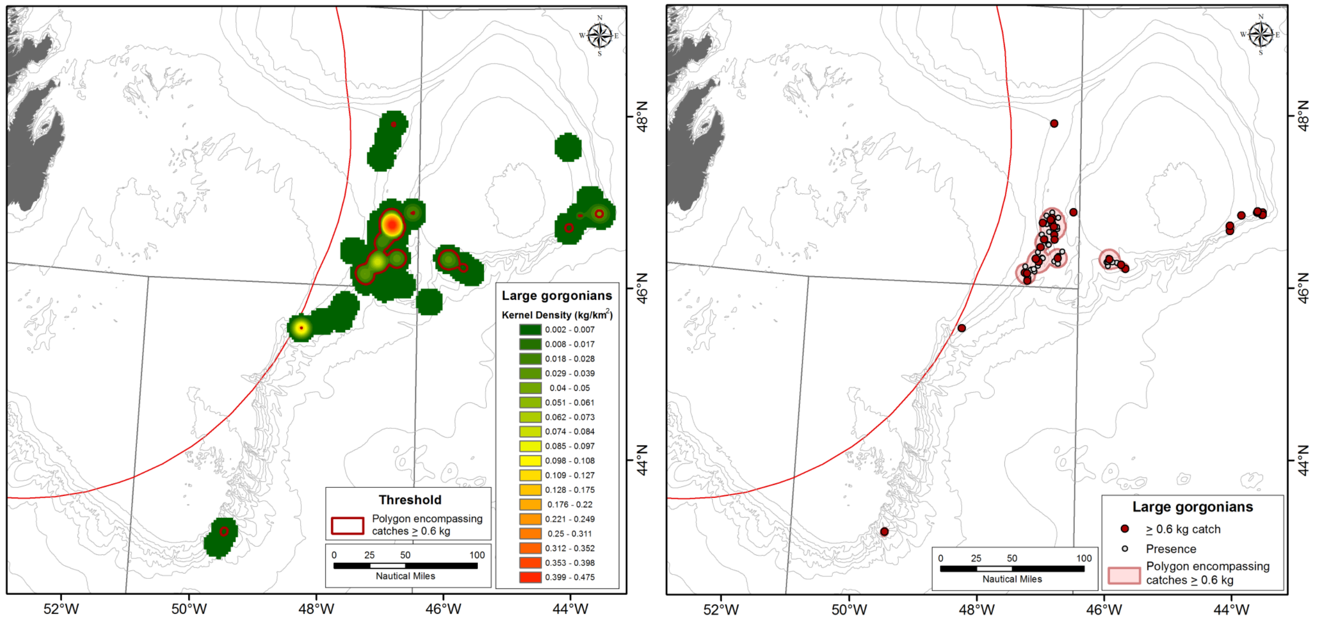

Figure 10. Left panel: Kernel density distribution of large gorgonian corals in the NAFO Regulatory area with the 0.6 kg density polygons defining significant concentrations superimposed in red. Right panel: The location of catches greater than 0.6 kg (red circle) and smaller coral catches (open circles) within the 0.6 kg density polygons defining the large gorgonian coral VMEs. doi:10.1371/journal.pone.0109365.g010

the spatial organization of the data which is critical to this application.

The outer boundaries of these VME areas are influenced by the search radius of the smoothing function and could in some instances, depending upon the distribution of the input data, extend beyond the VME habitat. For management purposes, the outer boundaries could be refined using targeted surveys, detailed surficial geology, multibeam bathymetry and/or overlay of species distribution models which incorporate environmental predictors such as depth. However, given the high vulnerability of these habitats to disturbance, the outer boundary could also be retained as a precautionary buffer zone from the significant adverse impacts of bottom trawling and dredging until further studies are made.

Although the VME areas delineated by our method utilize biomass thresholds to define boundaries for the calculation of area, the habitats are composed of catches from a wide range of biomass, including smaller catches that happen to fall amongst the larger ones (Figures 6, 8–10). These may represent areas thinned by fishing, areas of recruitment with smaller individuals dominating the catch, areas of different species composition within the VME taxon and/or minor catchability or effort differences among sets.

Of the VME indicator species/taxa in the NRA, the sponges represent the highest biomass in the research vessel catches [6] and form large-scale benthic habitats. Bell [24] divided the function of sponges in the benthic community into three different categories: 1) benthic-pelagic coupling (e.g., carbon and nitrogen cycling), 2) impacts on substrate (e.g., bio-erosion and sediment stabilization), and 3) habitat provision for other species (e.g., predation protection). The extensive sponge grounds in the NAFO NRA likely play important roles in ecosystem function, although only their role in provision of habitat has been directly looked at in any detail [18,19,25]. They are located in the deep water along the continental slopes in areas not generally targeted by the commercial fisheries, hence their persistence inside the fishing footprint.

The VME aggregations of sea pens, known as “fields”, provide important structure in low-relief sand and mud habitats where there is little physical habitat complexity (Figure 7). The dominant species in the study area do not retract into the sediment and so are vulnerable to trawl gear capture [8]. These fields provide refuge for small planktonic and benthic invertebrates [26], which in turn may be preyed upon by fish [27]. In the waters adjacent to the study area sea pens have been found to be associated with redfish larvae, raising the possibility that sea pen fields are important nursery areas for commercial fish species [28]. They also alter water current flow, thereby retaining nutrients and entraining plankton near the sediment [22]. The sea pen VMES occur in shallower water than the sponges on Flemish Cap and are adjacent to commercial fishing activity, which may already have impacted their abundance [8]. The KDE method would not be overly sensitive to local depletion of biomass caused by previous fishing, as long as some of the high catches are present in the grid cell either from historical data prior to the disturbance or through the inevitable patchy nature of the fishing effort leaving areas of high biomass undisturbed. The location of the sea pen field VMEs further suggests that the populations may be connected as they form a partial ring around the cap. Knowledge of connectivity patterns among these habitats is critical for the conservation of linked populations when not all habitats are protected.

The remaining VMEs addressed here, that is, the large and small gorgonian corals, are less prominent habitats in the NRA compared with the sponge grounds and sea pen fields. The functional roles that these corals play in the deep-sea benthic ecosystem are nevertheless important, especially for structure- or reef-forming corals, and have been well documented in terms of their benefits for other species [29]. Their structural complexity creates additional microhabitat that may be utilized by other organisms as refugia from predators, as spawning and nursery grounds, and as attachment substrate for sessile invertebrates [28,30,31]. Edinger et al. [32] examined the association between groundfish and 5 classes of corals, including large gorgonians, small gorgonians, sea pens and/or cup corals, soft corals, and the total absence of corals in adjacent Newfoundland waters. They found that of all five groups, groundfish species richness was highest in sets containing small gorgonians (*Acanella arbuscula* and *Radicipes gracilis*), highlighting the potential importance of this group as fish habitat. With the identification of coral and sponge VMEs in the NRA future research can be directed towards their ecosystem function so that a full assessment of the impacts of fishing activities can be made in accordance with UNGA 61/105.

Conclusions

Large-sized sponges, sea pens, and small and large gorgonian corals have been identified previously as vulnerable marine ecosystem indicators in the study area [2]. These large structure-forming taxa can be considered ecosystem engineers [33] as they modify the physical and chemical nature of their environment and provide habit for other organisms. Kernel density estimation (KDE) surfaces applied to research vessel trawl catches of vulnerable marine ecosystem indicators can be used to identify significant concentrations of biomass, thus serving to operationalize the definition of Structure Complexity under the FAO guidelines [1] for implementation of the 2006 United Nations General Assembly Resolution 61/105. The use of the area under the polygons of successive weight categories to identify global spatial thresholds for identifying the catch levels which separate aggregations from dispersed individuals is a novel approach. It has advantages over use of the point of maximum curvature in that it is derived from a geospatial context. We contend that the significant concentrations of sponges, sea pens, small and large gorgonian corals identified herein using this method, along with the supporting literature review of their ecological functions, qualify these areas as vulnerable marine ecosystems. The boundaries of these VME polygons can be refined using underwater cameras and species distribution modeling to delineate closed areas for management.

Acknowledgments

The authors acknowledge the contributions to the development of this work made through discussions with colleagues in the Northwest Atlantic Fisheries Organization (NAFO) Working Group on Ecosystem Science and Assessment (formerly the Working Group on the Ecosystem Approach to Fisheries Management) over the years 2009–2013, and the NAFO Scientific Council over that same time period.

Author Contributions

Conceived and designed the experiments: EK. Performed the experiments: FJM CL. Analyzed the data: EK FJM CL NO MS. Contributed to the writing of the manuscript: EK FJM CL LB VW NO MK AK MS.

References

1. FAO (2009) International Guidelines for the Management of Deep-sea Fisheries in the High Seas. FAO, Rome. 73p.
2. Fuller SD, Murillo Perez FJ, Wareham V, Kenchington E (2008) Vulnerable Marine Ecosystems Dominated by Deep-Water Corals and Sponges in the NAFO Convention Area. Ser No N5524. NAFO SCR Doc 08/22, 24pp.
3. NAFO (2008) Scientific Council Meeting 22–30 October 2008. Ser No N5594. NAFO SCS Doc 08/26, 32p.
4. Murillo FJ, Kenchington E, Sacau M, Piper DJW, Wareham V, et al. (2011) New VME indicator species (excluding corals and sponges) and some potential VME elements of the NAFO Regulatory Area. Ser No N6003. NAFO SCR Doc 11/73, 20 p.
5. NAFO (2012) Scientific Council Meeting –2012. Ser No N6072. NAFO SCS Doc 12/19, 215p.
6. Murillo FJ, Duran Munoz P, Cristobo FJ, Rios P, Gonzalez C, et al. (2012) Deep-sea sponge grounds of the Flemish Cap, Flemish Pass and the Grand Banks of Newfoundland (Northwest Atlantic Ocean): distribution and species composition. Mar Biol Res 8: 842–854.
7. Murillo FJ, Kenchington E, Gonzalez C, Sacau M (2010) The use of density analyses to delineate significant concentrations of Pennatulaceans from trawl survey data. Ser No N5753. NAFO SCR Doc 10/07, 7 p.
8. Kenchington E, Murillo FJ, Cogswell A, Lirette C (2011) Development of Encounter Protocols and Assessment of Significant Adverse Impact by Bottom Trawling for Sponge Grounds and Sea Pen Fields in the NAFO Regulatory Area. Ser No 6005. NAFO SCR Doc 11/75, 53 p.
9. Murillo F J, Durán Muñoz P, Altuna A, Serrano A (2011) Distribution of deep-water corals of the Flemish Cap, Flemish Pass, and the Grand Banks of Newfoundland (Northwest Atlantic Ocean): interaction with fishing activities. ICES J mar Sci 68: 319–332.
10. Fieberg J (2007) Kernel density estimators of home range: Smoothing and the autocorrelation red herring. Ecology 88: 1059–1066.
11. Downs JA, Horner MW (2012) Analysing infrequently sampled animal tracking data by incorporating generalized movement trajectories with kernel density estimation. Comput Environ Urban Syst 36: 302–310.
12. Benhamou S, Cornélis D (2010) Incorporating movement behavior and barriers to improve kernel home range space use estimates. J. Wildl Manage 74: 1353–1360.
13. Nelson T A, Boots B (2008) Detecting spatial hot spots in landscape ecology. Ecography 31: 556–566.
14. O'Brien SH, Webb A, Brewer MJ, Reid JB (2012) Use of kernel density estimation and maximum curvature to set Marine Protected Area boundaries: Identifying a Special Protection Area for wintering red-throated divers in the UK. Biol Conserv 156: 15–21.
15. Kenchington E, Cogswell A, Lirette C, Murillo Perez FJ (2009) The Use of Density Analyses to Delineate Sponge Grounds and Other Benthic VMEs from Trawl Survey Data. Ser No N5626. NAFO SCR Doc 09/6, 18p.
16. NAFO (2011) Report of the 3rd Meeting of the NAFO Scientific Council Working Group on Ecosystem Approaches to Fisheries Management (WGEAFM). Ser No N5868. NAFO SCS Doc 10/24, 75 p.
17. Beazley LI, Anstey LJ, Kenchington ER (2013) Summary of the Location of VME Indicators on the Flemish Cap Slope Based on *in situ* Benthic Imagery. Ser No N6274. NAFO SCR Doc 13/76, 18p.
18. Beazley LI, Kenchington E, Murillo FJ, Sacau M del M (2013) Deep-sea sponge grounds enhance diversity and abundance of epibenthic megafauna in the Northwest Atlantic. ICES J Mar Sci 70: 1471–1490.
19. Barrio Froján CRS, MacIsaac KG, McMillan AK, Del Mar Sacau Cuadrado M, Large P, et al. (2012) An evaluation of benthic community structure in and around the Sackville Spur closed area (Northwest Atlantic) in relation to the protection of vulnerable marine ecosystems. ICES J Mar Sci 69: 213–222.
20. Knudby A, Kenchington E, Murillo FJ (2013) Modeling the distribution of *Geodia* sponges and sponge grounds in the northwest Atlantic Ocean. PLoS ONE 8(12): e82306. doi:10.1371/journal.pone.0082306.
21. Knudby A, Lirette C, Kenchington E, Murillo FJ (2013) Species Distribution Models of Black Corals, Large Gorgonian Corals and Sea Pens in the NAFO Regulatory Area. Ser No N6276. NAFO SCR Doc 13/78, 17p.
22. Tissot BN, Yoklavich MM, Love MS, York K, Amend M (2006). Benthic invertebrates that form habitat structures on deep banks off southern California, with special reference to deep sea corals. Fish Bull 104: 167–181.
23. Rooper CN, Zimmermann M, Prescott MM, Hermann AJ (2014) Predictive models of coral and sponge distribution, abundance and diversity in bottom trawl surveys of the Aleutian Islands, Alaska. Mar Ecol Prog Ser 503: 157–176.
24. Bell JJ (2008) The functional roles of marine sponges. Estuar Coast Shelf Sci 79: 341–353.
25. Kenchington E, Power D, Koen Alonso M (2013) Associations of demersal fish with sponge grounds on the continental slopes of the northwest Altantic. Mar Ecol Prog Ser 477: 217–230.
26. Birkeland C (1974) Interactions between a sea pen and seven of its predators. Ecol Monogr 44: 211–232.
27. Krieger K J (1993) Distribution and abundance of rockfish determined from a submersible and by bottom trawling. Fish Bull 91: 87–96.
28. Baillon S, Hamel J-F, Wareham VE, Mercier A (2012) Deep cold-water corals as nurseries for fish larvae. Front Ecol Environ 10: 351–256.
29. Buhl-Mortensen L, Vanreusal A, Gooday AJ, Levin LA, Priede IG, et al. (2010) Biological structures as a source of habitat heterogeneity and biodiversity on the deep ocean margins. Mar Ecol 31: 21–50.
30. Fosså JH, Mortensen PB, Furevik DM (2002) The deep-water coral *Lophelia pertusa* in Norwegian waters: distribution and fisheries impacts. Hydrobiologia 471: 1–12.
31. Reed JK (2002) Deep-water *Oculina* coral reefs of Florida: biology, impacts, and management. Hydrobiologia 471: 43–55.
32. Edinger EN, Wareham VE, Haedrich RL (2007) Patterns of groundfish diversity and abundance in relation to deep-sea coral distributions in Newfoundland and Labrador waters. Bull Mar Sci 81: 101–122.
33. Jones CG, Lawton JH, Shachak M (1994) Organisms as ecosystem engineers. Oikos 69: 373–386.
